# Numerical value biases sound localization

**DOI:** 10.1038/s41598-017-17429-4

**Published:** 2017-12-08

**Authors:** Edward J. Golob, Jörg Lewald, Stephan Getzmann, Jeffrey R. Mock

**Affiliations:** 1 Department of Psychology, Tulane University, New Orleans, LA USA; 2 Program in Neuroscience, Tulane University, New Orleans, LA USA; 3 Faculty of Psychology, Ruhr University Bochum, D-44780 Bochum, Germany; 4 Leibniz Research Centre for Working Environment and Human Factors, Ardeystrasse 67, D-44139 Dortmund, Germany; 5 Present Address: Department of Psychology, University of Texas, San Antonio, USA

## Abstract

Speech recognition starts with representations of basic acoustic perceptual features and ends by categorizing the sound based on long-term memory for word meaning. However, little is known about whether the reverse pattern of lexical influences on basic perception can occur. We tested for a lexical influence on auditory spatial perception by having subjects make spatial judgments of number stimuli. Four experiments used pointing or left/right 2-alternative forced choice tasks to examine perceptual judgments of sound location as a function of digit magnitude (1–9). The main finding was that for stimuli presented near the median plane there was a linear left-to-right bias for localizing smaller-to-larger numbers. At lateral locations there was a central-eccentric location bias in the pointing task, and either a bias restricted to the smaller numbers (left side) or no significant number bias (right side). Prior number location also biased subsequent number judgments towards the opposite side. Findings support a lexical influence on auditory spatial perception, with a linear mapping near midline and more complex relations at lateral locations. Results may reflect coding of dedicated spatial channels, with two representing lateral positions in each hemispace, and the midline area represented by either their overlap or a separate third channel.

## Introduction

In speech recognition the meaning of sounds is decoded by mapping acoustic features onto lexical knowledge stored in long-term memory^1^. This basic problem of relating perceptual information to long-term memory is also evident in understanding visual object recognition, face recognition, and reading^2–4^. Categorizing the complex spectral and temporal dynamics of speech with respect to meaning is conventionally thought to be a one-way process: it starts with a sound and ends with meaning^5^. What if there is also a reverse influence? That is, can meaning influence the perceptual experience of basic speech sound features?

It is well-established that the context of a sentence can bias categorization of ambiguous speech sounds towards a meaning that fits the sentence^6^. This is a bias in the forward direction from first representing perceptual features, which are then used in the process of assembling representations of meaning. Sentence context has not been shown to modify perception of basic sound features such as location, loudness, and pitch. However, the potential for a reverse influence, which would be indicated by observations that long-term memory knowledge biases perception of a verbal stimulus, has scarcely been studied in any sensory modality. There is an older literature on how individual differences in valuation of the meanings of verbal stimuli may impact visual perceptual judgments^7,8^ and variability in auditory localization^9^. There is some evidence that long-term memory for the prototypical color of items, such as lemons being yellow, can influence color judgments^10–12^. For non-verbal stimuli there is also evidence that long-term memory for the structure of musical scales can influence pitch perception^13^.

In the current study we asked whether information in long-term memory can directly influence auditory spatial perception. We chose to use speech stimuli because they have an intimate relationship with long-term memory for lexical information that is necessary for speech-based communication. The Hansen *et al*. (2006), Olkkonen *et al*. (2008), and Shepard & Jordan (1984) studies all examined long-term memory information of features directly related to the perceptual task used for testing (i.e. color or musical pitch judgments). A potential concern with that approach is subject demand, because the direct relation between the task and lexical information may provide a subtle bias for subjects to meet experimental expectations.

Here, we took this approach a step further by probing the structure of long-term memory using an indirect, potentially implicit, relation between long-term memory for numbers and spatial perception. Convergent evidence from behavioral^14–20^, lesion^21^, and neuroimaging^22^ studies suggests a relation between number magnitude and spatial processing. The typical relation in Western cultures is that smaller numbers are associated with the left side of egocentric space and larger numbers have a bias towards the right^16,23^. Number magnitude can also influence visual perception in other ways, with similarities to what is observed with manipulation of luminance contrast^24^. Another example of a numeric influence other than a left/right bias is that reaction time in visual search shows an interaction between number magnitude and the size of digits^25^. Note that here we use the conventional term “number magnitude”, but strictly speaking number stimuli convey information on “numerical value”, which includes both magnitude and ordinality information.

Perhaps the best-known finding that relates numerosity to spatial representations is the SNARC effect^16^, which is present for visual as well as acoustic stimuli^26–28^. The basic SNARC effect is that in choice reaction time tasks, where response speed is emphasized, subjects respond faster to smaller numbers with their left hand and larger numbers with their right hand. The SNARC effect suggests an association exists between the left-to-right spatial layout of the hands and small-to-large number magnitudes within a given number range, and may relate to the number line used in Western cultures. Convergent behavioral and neurophysiological evidence suggests that the SNARC effect is attributable to a later response selection stage of cognitive processing^17,29,30^. Thus, the SNARC effect relies on differences in speeded performance to understand how numerosity influences response selection.

In the above studies showing relations between number magnitude and space, participants made responses to numbers but few examined whether number magnitude had an influence on how subjects perceived the number stimuli. The reason is that reaction time measures supply information about the time needed for information processing but they do not tell us about the subject’s conscious experience of having perceived a given stimulus. The same issue also applies to other performance measures such as accuracy. Conventionally, many cognitive scientists make a coarse distinction between early stages of stimulus processing that involve extracting information about stimulus features (e.g. shape, color, intensity, pitch) in relation to long-term knowledge, from post-perceptual processes on how the perceptual information is used (e.g. selecting a particular response, memory encoding, adjustments to attention control and executive function processes, etc.)^31^. Studies of the SNARC effect require speeded responses, and usually target later stages of processing that index response selection. In contrast, the present study examines long-term memory influences on early perceptual stages, and focuses on perceptual judgments about the location of sounds rather than reaction time.

The purpose of the current study was to examine whether number magnitude influences spatial processing at other stages besides response selection. We chose a very basic perceptual process - sound localization. The primary measures in our study used psychophysical methods to index perceptual judgments, and these measures are independent of response speed (see Methods). The main interest is whether the subject’s perception of sound location relates to number magnitude. A previous study from our labs showed that numbers can prime later spatial judgments of a white noise stimulus^32^. However, judgments concerning the location of the preceding number were not examined. A crossmodal study supported the rationale of the current study by showing that visually-presented numbers can influence loudness judgments^33^. In this study we conducted a series of experiments using different protocols, languages, and measurement methods to test the idea that auditory spatial perception along the left-right axis is influenced by long-term memory for number magnitude.

## General Methods

### Participants and audiometric testing

A total of 105 participants were tested in 4 experiments. Participants were recruited from Ruhr University Bochum (Experiment 1) and Tulane University (Experiments 2–4). Experiment 1 relied on a self-report of no hearing problems. In Experiments 2, 3, and 4 participants were tested for hearing thresholds using an audiometer (0.5–8.0 kHz), and all had hearing thresholds within normal limits (0.5–4.0 kHz; ≤25 dB threshold; ≤10 dB difference between ears). Prior to inclusion in the study all subjects gave their written informed consent for protocols approved by Ruhr University Bochum and Tulane University review boards. The experiments followed standards of the 1964 Declaration of Helsinki. Subjects received course credit for their participation.

### Statistical analysis

Data were analyzed using analysis of variance (ANOVA) tests, and included factors of number magnitude and the location of acoustic number stimuli. The levels in these factors differed among experiments, and will be specified in each experiment’s section below. Significance was defined as *p* < 0.05, and effect sizes were calculated using ${\eta }_{p}^{2}$. In Experiment 1 the choice of running 25 subjects was based on our previous experience in similar studies (cf. Ocklenburg, Hirnstein, Hausmann, & Lewald, 2010^34^), and included the likelihood of having outlier subjects that would be excluded from the analysis. For Experiments 2–4 there were no previous studies to provide an effect size for power analysis. We planned to also use 25 subjects per experiment as in Experiment 1, but 20 were used because the results were clear and replicable with a smaller sample size. As the data were being collected for Experiment 2 it became clear by informal observation that there was a very strong effect. The decision to stop at 20 subjects was made after running about 15 subjects. At this point 13/15 subjects had a more positive point of subjective equality for number 1 vs. 9, with a linear progression between these numbers. In the end, as will be presented below, 18/20 subjects in Experiment 2 showed this effect; a strong result for a behavioral study. Adding more subjects did not appear necessary, which was validated by the consistent results showing number bias in each of the four experiments.

## Experiment 1

### Method

#### Participants

Twenty-five subjects participated in Experiment 1. Three subjects were excluded as outliers (defined as >1.5 times the interquartile range above the third quartile and 1.5 times the interquartile range below the first quartile), due to their deviation in mean constant error in localization of control stimuli (see below). A total of 22 right-handed subjects were included in the analysis (mean age 25 ± 3; age range 19–32 years; 11 men, 11 women). Subjects were tested in a dark, sound-proof, and anechoic room (for details see Guski, 1990^35^).

#### Materials and apparatus

Auditory stimuli on each trial were presented from one of six full-range loudspeakers (Visaton SC 5.9, Visaton, Haan, Germany; 5 × 9 cm2) located at 30°, 18°, and 6° to the left and right of the subject’s median plane. The loudspeakers were part of a larger semicircular array of 91 loudspeakers, each at a distance of 1.5 m from the center of the subject’s head (for details, see Lewald, Wienemann, & Boroojerdi, 2004^36^). Custom-fitted chin, forehead, and occiput rests were used to minimize head movements.

Two types of speech stimuli were used as targets for sound localization in the experiment: digits that were replayed as recorded and, as a control, the same digits were played backwards. When presented forward (as recorded) the target numerals were perceived normally, but when reversed they were a speech-like sound that was not perceived as a number. The reverse and forward sounds differed in envelope, but not in duration, level, and frequency content, and were presented at 68 dB (A). The stimuli were the German numerals “eins” (one), “drei” (three), “acht” (eight), and “zehn” (ten) spoken by a native male German speaker and digitally recorded in an anechoic setting at a sampling rate of 48 kHz. These numbers were chosen because their magnitudes were near the extremes of the digit range, were monosyllabic, and had comparable durations (mean 560 ms; range 500–600 ms).

#### Procedure

In each trial, the subject first heard a sound from one of the six speaker locations, then directed a hand pointer (a swivel rod mounted in front of the subject in the trunk median sagittal plane) to the perceived location of the target sound, and lastly pressed a button (mounted on the upper side of the pointer) to record the azimuth angle of the pointer (for details of this pointing task, see Lewald, Dörrscheidt, & Ehrenstein, 2000^37^). The subject had to press the response button within 4.1 s after stimulus onset. Otherwise the trial was automatically repeated at the end of the experimental block. Trials were presented with a constant intertrial-interval of 5.0 s. Four blocks of 144 trials were given (576 trials total), which were separated by short rest breaks (<10 min total). Combinations of sound presentation (forward, reversed), digit (1, 3, 8, 10), and location (6 positions) were counterbalanced within blocks, and successive stimuli from the same location did not occur.

In Experiment 1 the dependent variable was the location of pointing responses to digit stimuli normalized to presenting the same digit in reverse. The pointer was controlled with both hands, and subjects generally took several seconds to align the pointer with the perceived sound location. Thus, this task did not evaluate speeded two-choice response selection, as is typical in experiments on the SNARC effect. Pointing responses were normalized to compensate for variability between subjects as well as a general localization bias toward more lateral positions when pointing to eccentric sounds (Lewald *et al*., 2000^37^). Normalization was done by subtracting angular localization values in the reverse condition from angular localization when sounds were played forward. Relative to reverse presentation, negative values indicated that forward presentation was perceived to the left of reversed stimuli, and positive values indicated a perceived location to the right. The normalization measure was separately calculated for each number, and target position.

### Results

**Figure 1:**
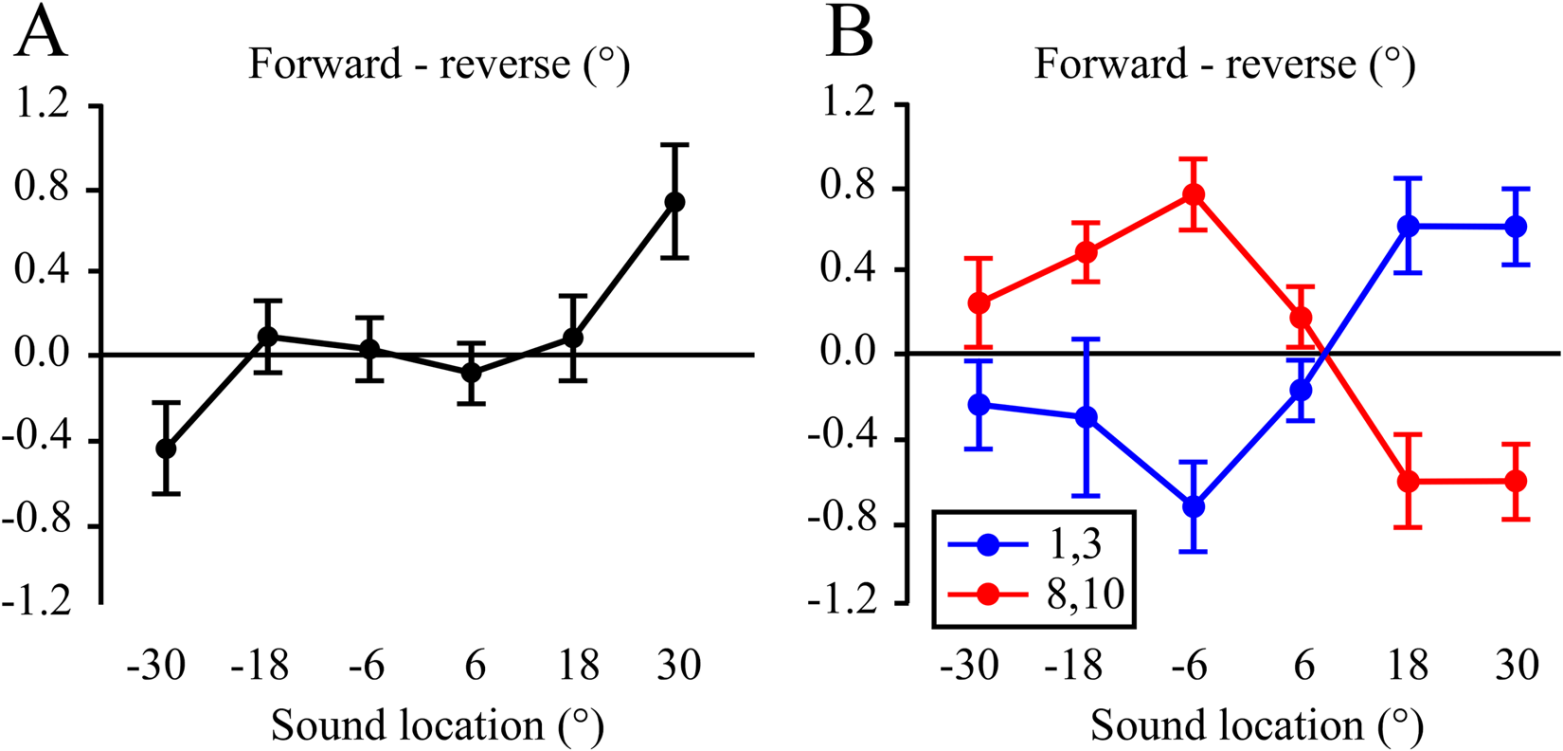
Experiment 1 results. Constant error in sound localization as a function of stimulus type (forward, reversed) and target location. (**A**) Normalized constant errors, calculated by subtracting values for reversed from those for forward target presentation. (**B**) Comparison of normalized constant errors for localization of numbers that were smaller (1, 3) vs. larger (8, 10) in magnitude. Normalization was performed by subtracting constant errors for reversed control sounds from constant errors for the semantic (forward) stimuli. Negative values indicate that forward stimuli were perceived to the left of reverse stimuli, and positive values indicate that forward stimuli were perceived to the right of reversed stimuli. (**B**) Plot of smaller and larger numbers as a function of hemispace. (**C**) Comparison of smaller and larger numbers as a function of eccentricity. (**D**) Plot combining all numbers as a function of stimulus eccentricity.

We first compared the overall localization of forward vs. reversed speech as a function of location by collapsing across digit magnitude. The normalized localization measures (forward – reverse) at the six target locations are shown in Fig. 1A. Values around zero indicated no overall differences between the forward and reversed targets in the range of ±18°. However, at the ±30° locations the forward targets were perceived to have a more eccentric location as compared to reversed targets. This impression was confirmed by running a 2 (stimulus type: forward, reversed) ×6 (location) ANOVA test, which had a significant stimulus × location interaction (*F*_(5,105)_ = 3.7; *p* < 0.03; ${\eta }_{p}^{2}$ = 0.15).

The hypothesis that small numbers bias judgments to the left and large numbers to the right was tested by comparing localization of small (1, 3) vs. larger (8, 10) numbers. The normalized measures for each subject were corrected by their overall localization values at each location in Fig. 1A. This had little effect on data for the ±6° and ±18° locations, but for the ±30° locations it moved the overall values toward the midline.

A plot of normalized localization as a function of number magnitude and loudspeaker location is shown in Fig. 1B. A 2 (magnitude: small, large) × 6 (location) ANOVA test revealed a significant interaction of magnitude × location (*F*_(5,105)_ = 9.0; *p* < 0.001; ${\eta }_{p}^{2}$ = 0.30). This indicates that localization was different for the small vs. large numbers, but that the specific direction of the effect depended on location. The results were as predicted for the left hemispace and +6°, as localization of the smaller numbers was shifted to the left of the larger numbers. In the right hemispace, particularly +18° and +30°, the opposite pattern was seen: there was a localization bias to the right for small numbers and the left for larger numbers. Thus another interpretation of the results is that small numbers induced an eccentric localization bias and larger numbers had a central bias.

## Experiment 2

Experiment 2 used a different approach to test the hypothesis that number magnitude biases sound localization, and also addressed several limitations of Experiment 1. Experiment 1 showed the expected left-right “number line” bias at the most central locations, but the interpretation was more ambiguous at lateral locations. To clarify the midline effects we next had participants judge the location of numbers presented near the midline. The forward and reversed stimuli are a good match in terms of basic stimulus features, but reversing the stimulus does change the envelope which, conceivably, may differ for small vs. large numbers and affect localization. In Experiment 2 the number stimuli were spoken in English by a different person, and we did not compare forward vs. reversed sounds. The pointing task in Experiment 1 requires a transformation from an auditory head-centered spatial reference frame to a body or hand-related reference frame needed to control the swivel hand pointer. It also takes several seconds to move the pointer to its final position on each trial, which requires storage of the original sound location in short-term memory, a process that can result in systematic errors in localization^38^. For other methods used to study movement trajectories and their relation to numeric cognition see^39^. The differences in localization between small and large numbers, as well as the complex pattern of results could relate to reference frame transformations and/or short-term memory demands.

To circumvent these issues, in Experiment 2 subjects listened to sounds presented near the midline and were only asked to judge if the sound was to the left or right of midline. The response was a button press, which simplified the reference frame issue, and responses were rapid (~500–600 ms), which reduced concerns about short-term memory influences. The procedure was a 2-alternative forced choice test, which is a standard psychophysical method.

### Method

#### Participants

Twenty young adults participated in the main experiment (mean age 23 ± 3 years; age range 18–30; 16 right handed; 6 men, 14 women). A control group that performed the same task but heard white noise stimuli rather than numbers was also tested (*n* = 20, age = 20 ± 1 years; age range: 18–22; 18 right handed; 9 men, 11 women). The purpose of the control group was to provide a comparison condition where subjects judged the location of sounds that did not convey any speech information.

#### Apparatus and materials

Experiment 2 focused on whether number magnitude can bias localization in the horizontal plane for stimuli presented close to midline. English digits from one to nine spoken by an adult male were first digitally recorded at a sampling rate of 44.1 kHz. Stimuli were presented using insert earphones (mean duration 440 ms; range 290–570 ms)(Etymotic Research, Elk Grove Village, IL USA) at ~60 dB nHL. Each of the recordings of the nine digits was transformed into nine files that corresponded to nine locations in virtual space (0° midline, ±2°, ±4°, ±6°, ±12° to the left and right). The virtual sound locations were created by applying the appropriate differences in interaural time, level, and head related transfer functions to elicit a percept of the digit at the intended location (SLAB software, National Aeronautics and Space Administration). By convention negative location values are to the left of 0° (midline) and positive locations are to the right. The algorithms mimic how the auditory system uses these same cues (time and level differences, head-related transfer functions) for sound localization under natural conditions^40^. Although there are individual differences in the precise set of cues for spatial hearing, subjective impressions of participants as well as the behavioral data verified that the sounds were perceived at the intended locations (see Results section).

#### Procedure

For the task itself, participants listened to a sequence of numbers and made a two-alternative forced choice by judging whether each number was to the left or right of their subjective midline. Midline was explained as the location straight ahead relative to the middle of their head, and all subjects understood the concept. Participants indicated their choice by pressing one of two buttons with their left/right thumb. Subjects were instructed to respond at a pace that ensures that they provide accurate judgments of left or right stimulus location, and to not respond rapidly. The stimulus onset asynchrony (SOA) was fixed at 2.4 sec, which allowed ample time to respond and a comfortable, predictable presentation rate. The sequence was pseudorandom with the restrictions that each number was presented 18 times and the same number was not delivered on consecutive trials. Each block of trials contained 162 stimuli, and five blocks were tested for each subject. The subjects in the control group performed the same task but heard white noise stimuli instead of numbers (0.1–10,000 Hz, 440 ms duration to match the duration of number stimuli, ~60 dB nHL).

#### Data analysis

The main dependent variable was the point of subjective equality (PSE), which is defined as the location where a subject is equally likely to perceive a stimulus to the left or right. Note that the PSE is determined by the pattern of left or right judgments; reaction time is not considered. The PSE was derived by fitting a Weibull psychometric function to the percentage of “right” judgments as a function of virtual stimulus location (−12° to +12°). As is typically found in perceptual judgments of parametrically varied stimulus features, the function was sigmoidal, with small values for locations on the far left side, rising steeply at intermediate locations, and reaching a near asymptote for locations on the far right. These functions were fitted to each of the nine numbers (described in detail below). The psychometric function had four parameters: the PSE, the slope of the psychometric function, and minimum (gamma), and maximum (lambda) parameters at the asymptote of 0 and 100% right responses. Gamma and lambda were fixed at 0.05; results with these parameters at 0.01 yielded the same patterns of results reported below. Psychometric slope data were not presented here because the hypothesis of interest concerned the subject’s judgment of sound location rather than details of the shape of the psychometric function.

Secondary analyses examined sequence effects across trials and reaction times. The analysis of sequence effects tested for whether the location of the number stimulus on the previous trial affected how the next number was localized. The location of the number on the prior trial was noted and later entered into the ANOVA tests as a factor (significance = p < 0.05, two-tailed). Preliminary analyses in Experiments 2, as well as 3 and 4, that included number magnitude as a factor showed that number magnitude did not influence sequence effects, and consequently the analyses of sequence effects was collapsed across magnitude. Reaction time data were included to show whether the well-known distance effect was present. The distance effect occurs when participants make two-choice perceptual judgments along a continuous dimension (e.g. space, pitch, line length, etc.) and reaction times progressively decrease away from the criterion value that is used to categorize the two responses^41^.

### Data availability

The datasets generated during and/or analyzed during the current study are available from the corresponding author.

### Results

**Figure 2:**
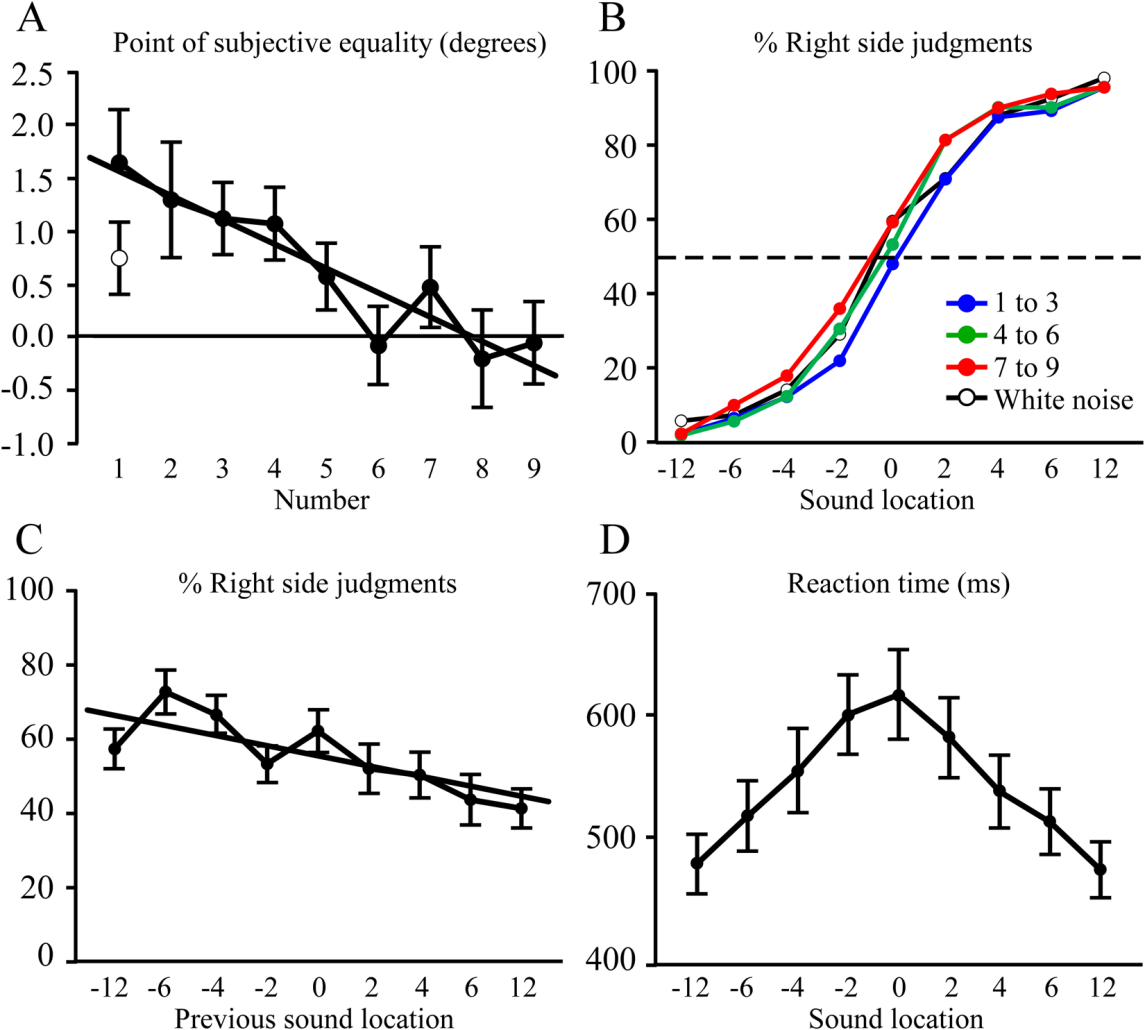
Experiment 2 results. (**A**) Point of subjective equality (PSE) plotted as a function of number magnitude. There was a significant negative linear relationship between PSE and number magnitude, indicated by the regression line. A separate control group, indicated by the open circle, performed the same task using a white noise stimulus rather than numbers. The controls had a PSE approximately midway between the numbers 1 and 9. (**B**) Psychometric functions of “% right” judgments as a function of sound location. For illustration numbers are shown in three groups (1–3, 4–6, 7–9), as well as the white noise control group. The 50% PSE is indicated by a horizontal dotted line. (**C**) Sequence effects are shown by plotting “percent right” judgments as a function of the location of the previous number. There was a significant effect of sequence as judgments were inversely related to the location of the previous number. (**D**) Reaction time vs. sound location. There was a significant distance effect, indicated by progressive reductions in reaction time as sound location was farther from the midline.

The PSE measures for each individual number are shown in Fig. 2A. For illustration of the overall psychometric functions, the numbers were evenly grouped by magnitude (1–3, 4–6, 7–9) in Fig. 2B. The PSE had a systematic shift depending on number magnitude, with large numbers having the left-most PSE, medium numbers were intermediate, and the PSE for small numbers was farthest to the right. Note that PSE shifts to the left mean that a larger range of locations is perceived as being to the right of true midline, and vice versa for a rightward shift in PSE. Thus the pattern matches what is expected if number magnitude slightly expanded the space associated with a given number’s magnitude. Small numbers had added space perceived as being to the left of midline, which pushed the judgment of midline to the right. Larger numbers biased more of the tested space to the right, and consequently the PSE was shifted to the left for large numbers. For intermediate numbers left/right judgments were fairly accurate relative to the true sound location.

We quantitatively examined the PSE measure as a function of number magnitude (1–9) with a one-way ANOVA test. There was a significant effect of location (*F*_(8,152)_ = 6.0; *p* < 0.001; ${\eta }_{p}^{2}$ = 0.24), that was well-fit by a linear model where increases in number magnitude were accompanied by decreases in PSE (*F*_(1,19)_ = 28.7; *p* < 0.001; ${\eta }_{p}^{2}$ = 0.60, r^2^ = 0.87). The PSE of the white noise control group was comparable to the intermediate numbers, and was in between the small and large number PSEs (Fig. 2A).

The overall PSE when collapsed across all numbers for most subjects was very close to the intended 0° location of the virtual stimuli (mean = 0.78° SD = 1.30°), which was also seen in the control judgments of white noise (mean = 0.63°, SD = 1.48°). Given the importance of where subjects placed their midline criterion, we decided to examine whether there were systematic effects of the stimulus’ location in the previous trial on judgments of the stimulus in the current trial (termed “sequence effects”). There were not enough trials to calculate psychometric functions for each of the seven locations of stimuli before a current trial. Instead we used the percentage of “right” side judgments as the dependent variable.

The results from the sequence effect analysis are plotted in Fig. 2C. Preliminary analyses showed no significant effects of number magnitude, so the analysis collapsed across all numbers. The percentage of right side judgments was examined at the 0° location as a function of the sound location in the previous trial. Sounds at midline were selected because they are ambiguous in terms of left or right, which would best reveal any sequence effects.

A one-way ANOVA test with the factor of previous location (−12°, −6°, −4°, −2°, 0°, +2°, +4°, +6°, +12°) had a significant effect (*F*_(8,144)_ = 6.1; *p* < 0.001; ${\eta }_{p}^{2}$ = 0.25). The effect of location indicated a linear relationship between sound location on the previous trial and the current trial’s left/right judgment (*F*_(1,18)_ = 13.8; *p* < 0.01; ${\eta }_{p}^{2}$ = 0.43). When the previous sound was on the left subjects were more likely to judge the ambiguous 0° stimulus to be to the right of midline, and vice versa for sounds on the right. This suggests that each stimulus induces a small shift in the PSE from true midline towards the sound’s location. The shift biases left/right judgments away from the side of the prior sound, and persists at least until the next number stimulus is delivered.

Although response speed was not emphasized, participants responded promptly to the numbers (mean = 545 ± 28 ms, range = 347–816 ms; Fig. 2D). This provided an opportunity to supply convergent evidence along with the overall PSE to define the location that subjects thought was midline, and is based on prior work on the “distance effect”. Here, a one-way ANOVA of location (1–9), collapsed across number magnitude, showed that reaction times were longest at 0° and progressively decreased at farther locations on either side (*F*_(8,144)_ = 27.1; *p* < 0.001; ${\eta }_{p}^{2}$ = 0.60), indicating that subjects used the actual location of 0° as the basis for their left/right judgments.

## Experiment 3

The third experiment had two main purposes. The first was to replicate the findings of Experiment 2 where left/right judgments were defined with reference to the subjective sense of midline. The second purpose was to compare left/right judgments at midline with vs. without a preceding cue that was presented at midline. The cue was used as a reference point for the same left/right judgment task. Developing this method would allow for the cue to be presented at locations other than midline (see Experiment 4). The only difference between the cued condition and the task in Experiment 2 was that a white noise stimulus was presented at midline 1.0 s before the onset of the number stimulus. Given the finding from Experiment 2 that the previous stimulus position affected localization of the current stimulus, we decided to compare left-right judgments at midline with vs. without the prior cue because the cue could have affected left/right judgments of the subsequent number.

### Method

#### Participants

Twenty new young adults participated in Experiment 3 (mean age 19 ± 1 years; age range 18–21; 18 right handed; 5 males and 15 females), and received the same screening procedures as in Experiment 2.

#### Materials and apparatus

Experiment 2 used a two-alternative forced choice that used the subject’s perception of midline (more precisely, the median sagittal plane of the head) as a reference for making their response choice. Although the sense of midline is intuitive and fairly accurate, this approach alone would not work well for lateral locations which lack such an intuition. Here we used a white noise cue (0.1–10 kHz; 440 ms duration; ~60 dB nHL) to indicate the location that will serve as the reference for two-alternative (left vs. right side) judgments of the location of the subsequently presented number. Cue duration was set to match the mean duration of number stimuli. The cue was followed 1.0 s later by the digit number, and as in Experiment 2 the subject judged whether the number was located to the left or right of the cue. The inter-trial interval between onset of the number and onset of the cue for the next trial was 2.0 s.

#### Procedure

Experiment 3 was designed to validate the cue-number method by comparing it to the midline judgment used in Experiment 2. In Experiment 4 the cue-number will be used to test localization judgments at lateral locations. Participants were given 3 blocks of the cued paradigm described above and 3 blocks of midline judgments without the cue, as in Experiment 2. Experiment 3 also functioned as an opportunity to replicate the results from Experiment 2. The order of cue and no-cue tasks was counterbalanced across subjects.

### Results

**Figure 3:**
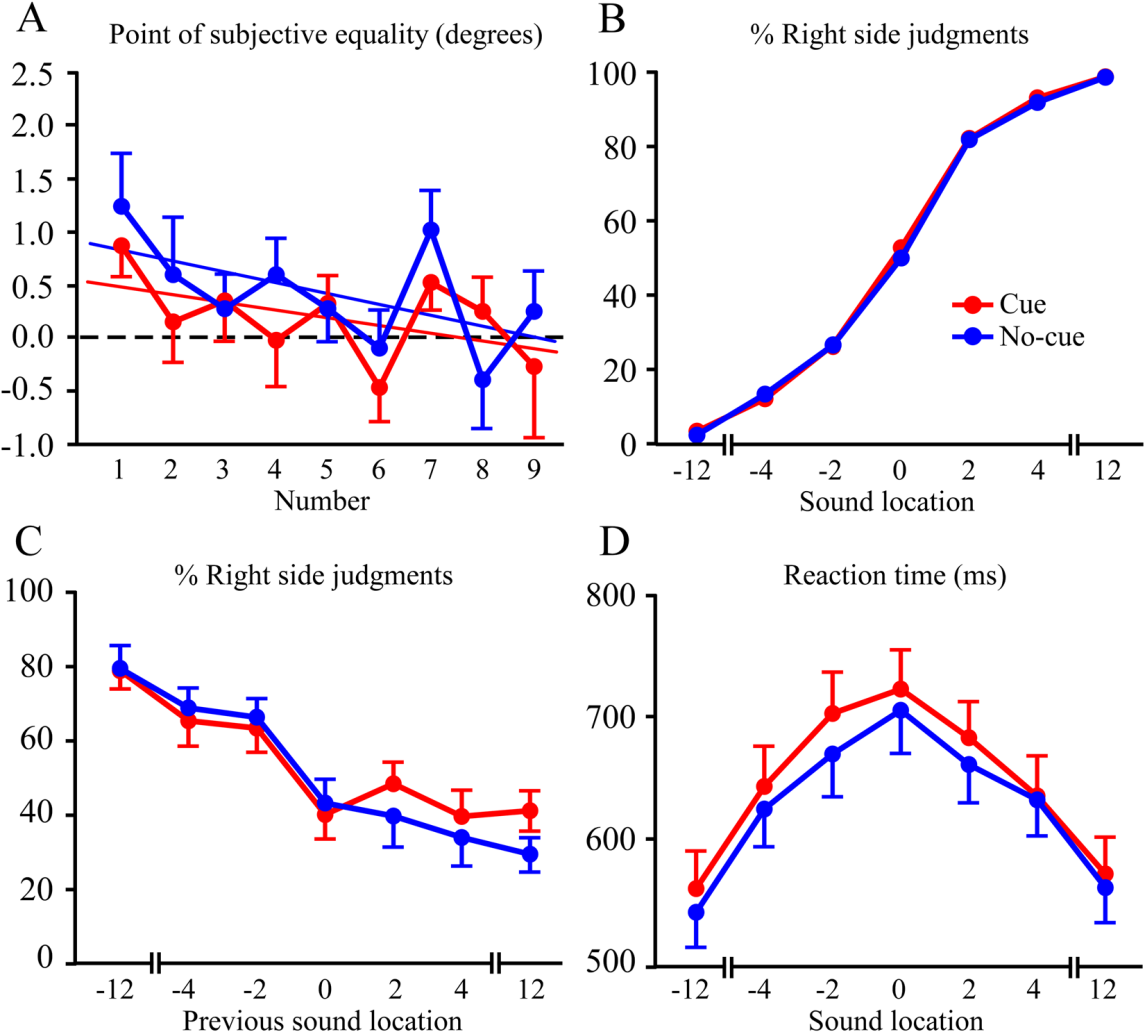
Experiment 3 results. (**A**) Point of subjective equality (PSE) plotted as a function of number magnitude in the cue and no-cue conditions for midline judgments. Overall, PSE significantly decreased with increases in number magnitude. (**B**) Overlapping psychometric functions for % right judgments in the cue and no-cue conditions. (**C**) Sequence effects for % right judgments as a function of previous sound location. In both conditions there was a significant negative association between the likelihood of a “percent right” judgment and location of the previous trial’s number. (**D**) Plot of reaction time vs. sound location. There was a significant distance effect in both the cue and no-cue conditions, shown by decreases in reaction time with greater distance from midline.

The PSE measures as a function of number magnitude are shown in Fig. 3A for the cued and uncued conditions. The psychometric functions for cue and no-cue conditions were nearly identical (Fig. 3B). The mean PSE values collapsed across number magnitudes were comparable among conditions, with 0.55 (SD = 1.26) in the no-cue condition and 0.33 (SD = 1.18) in the cued condition.

A 2 (condition) × 9 (number magnitude) ANOVA test of PSE measures showed a significant effect of number magnitude (*F*_(8,152)_ = 2.8; *p* < 0.05; ${\eta }_{p}^{2}$ = 0.13). This was due to the PSE changing relative to true midline (0°) as number magnitude increased, from having a bias towards the right to a bias towards the left. The interaction of condition × number magnitude was not significant, although Fig. 3A suggests that the effect may be stronger in the no-cue condition (no-cue *r* ^2^ = 0.30, cue *r* ^2^ = 0.24).

Sequence effects are shown in Fig. 3C for the cue and no-cue conditions, and as in Experiment 2 the results were collapsed across individual number magnitudes. A 2 (condition) × 7 (previous location) ANOVA test with the dependent variable of percentage of “right” judgments indicated a significant effect of previous trial location (*F*_(6,114)_ = 30.2; *p* < 0.001; ${\eta }_{p}^{2}$ = 0.61). There was a strong linear relationship between sound location on the previous trial and the left/right judgment in the current trial (*F*_(1,19)_ = 114.1; *p* < 0.001; ${\eta }_{p}^{2}$ = 0.86). When the previous sound was on the left subjects were more likely to judge the 0° stimulus to be to the right of midline. The opposite bias was seen for previous sounds on the right side. There were no significant effects of condition or the condition × previous location interaction.

The analysis of reaction times used a 2 (condition) × 7 (sound location) ANOVA, and as with Experiment 2 there was a significant effect of location (*F*_(6,114)_ = 49.6; *p* < 0.001; ${\eta }_{p}^{2}$ = 0.73). The slowest reaction times were at 0°, with symmetrical reaction time decreases with greater distance on either side of midline (Fig. 3D).

In summary, the no-cue condition replicated the finding in Experiment 2 by showing that the PSE systematically varies with number magnitude. The PSE was oriented to the right of midline for small numbers, and progressively shifted leftward past midline with increases in number magnitude. Sequence effects from Experiment 2 were also replicated, indicating that irrespective of number magnitude left/right judgments for a number at 0° were biased in the opposite direction of the previous number’s location. This pattern was seen even when a cue stimulus was interposed between numbers. Reaction time data also replicated Experiment 2, and showed that reaction times were slowest for numbers at 0°, and sped up with greater distance from the midline.

## Experiment 4

Experiment 4 used the same basic cue-target task employed in Experiment 3. The only difference was that location judgments relative to lateral locations were tested by presenting the cue at one of two lateral locations (±18°, in separate blocks). Lateral cues were used because, unlike midline, subjects do not have a precise sense of where ±18° locations are positioned in egocentric space. A clear understanding of the reference point is needed for left/right judgments over the small range of locations that were tested (±12° relative to the cue location). Selection of ±18° as the locations for making left/right judgments was based on the results of Experiment 1, in which the interaction of number magnitude and location became more complex with more eccentric locations. The cue location was constant within a block to avoid the influence of attentional cueing effects^42^, which may also encourage a more consistent localization of the cue. The order of blocks having a left or right location cue was counterbalanced across subjects.

### Method

#### *Participants*, *Materials and apparatus*, *and Procedure*

A new set of participants was run (*n* = 20, mean age 19 ± 1 years; age range 18–21; 17 right handed; 5 males and 15 females). The procedures in Experiment 4 were the same as in Experiment 3 except that the cue was presented, in separate blocks, at ±18° from midline. As in Experiment 3, participants were told to use the cue location as a reference point to determine their left/right judgments of the following number.

### Results

**Figure 4:**
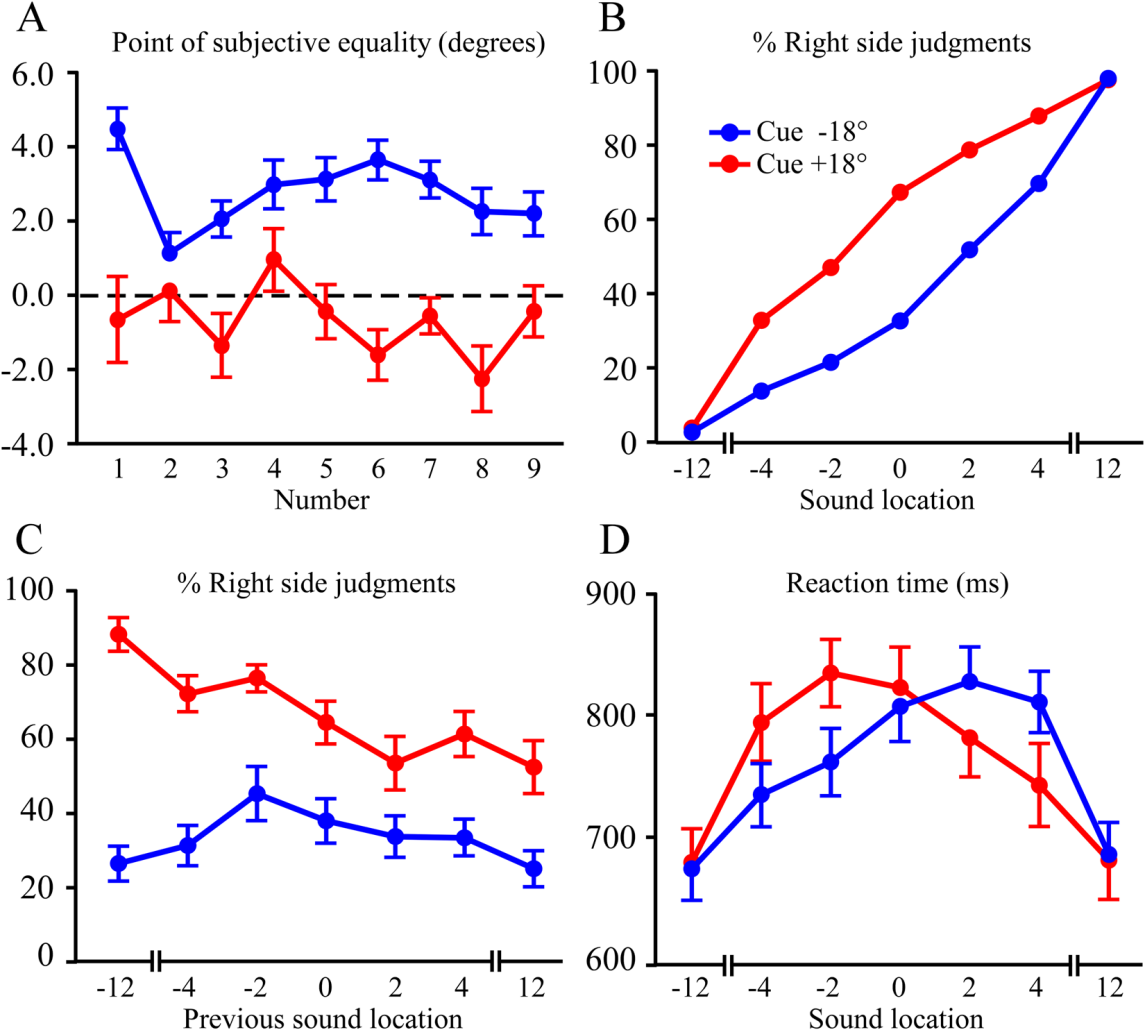
Experiment 4 results. (**A**) PSE plotted against number magnitude in the cue and no- cue conditions at lateral cued locations (±18°). There was a significant effect of number magnitude  when the left (−18°) or right side (+18°) were cued. (**B**) Psychometric functions of “% right” as judged with respect to the left (−18°) and right (+18°) cued locations. In the cue −18° condition the PSE was significantly to the right of 0°. (**C**) Plot of percent right judgments as a function of previous number location. There were significant sequence effects. Relative to the −18° cue, cueing on the right side had overall more “right” judgments and the profile was more linear. (**D**) Reaction time vs. sound location in the cue left and cue right conditions. For both conditions there were significant distance effects, and longer reaction times were skewed in the direction of midline.

The PSE as a function of location relative to the cues is shown in Fig. 4A, and the psychometric functions for left and right cues are presented in Fig. 4B. The mean PSE values for all number magnitudes were 2.83° (SD = 1.50) in the left cue condition and −0.55° (SD = 2.47) in the right cue condition. A 2 (cue location) × 9 (number magnitude) ANOVA test revealed significant effects of cue location (*F*_(1,19)_ = 40.5; *p* < 0.001; ${\eta }_{p}^{2}$ = 0.68) and number magnitude (F_(8,152)_ = 3.8; *p* < 0.01; ${\eta }_{p}^{2}$ = 0.17). The main effects were qualified by a significant cue location × number magnitude interaction (*F*_(8,152)_ = 4.3; *p* < 0.01; ${\eta }_{p}^{2}$ = 0.19). Follow-up one-way ANOVA tests of number magnitude were conducted separately for the left and right cue conditions. There was a significant effect of number magnitude in both the left (*F*_(8,152)_ = 5.3; *p* < 0.001; ${\eta }_{p}^{2}$ = 0.22) and right cue conditions (*F*_(8,152)_ = 3.3; *p* < 0.02; ${\eta }_{p}^{2}$ = 0.15), but the profile across number magnitudes was different. The left cue condition had a large rightward shift in PSE for #1, and a drop for #2 that gradually rose and then decreased for #3–#9. In the right cue condition the PSEs were irregular, with perhaps a decrease in PSE between #4 and #8. These complex results will be covered in more detail in the Discussion.

The sequence effects collapsed across number magnitude are shown in Fig. 4C for both cue locations. A 2 (condition: cue left, cue right) × 7 (previous location) ANOVA on the percentage of “right” judgments had main effects of condition (*F*_(1,19)_ = 46.3; *p* < 0.001; ${\eta }_{p}^{2}$ = 0.71), previous location (*F*_(6,114)_ = 6.5; *p* < 0.001; ${\eta }_{p}^{2}$ = 0.25), and an interaction of condition × previous location (*F*_(6,114)_ = 6.7; *p* < 0.001; ${\eta }_{p}^{2}$ = 0.26). As above, the previous location effect was a linear association between sound location in the previous trial and the left/right judgment in the current trial (*F*_(1,19)_ = 12.3; *p* < 0.01; ${\eta }_{p}^{2}$ = 0.39).

A 2 (cue location) × 7 (sound location) ANOVA test was used to assess reaction times (Fig. 4D). There was a significant effect of location (*F*_(6,114)_ = 30.0; *p* < 0.001; ${\eta }_{p}^{2}$ = 0.61) and a cue × sound location interaction (*F*_(6,114)_ = 10.6; *p* < 0.001; ${\eta }_{p}^{2}$ = 0.36). For simplicity the locations (−12° to +12°) in Fig. 4D are expressed relative to each respective cue, which was ±18° from true midline. Unlike Experiments 2 and 3, here the peaks of the reaction time × location curves were not symmetrical. Instead they were displaced towards the midline for each cue (i.e. 2° farther to the right for the left cue and 2° farther to the left for the right cue). This suggests that true midline has an attractive bias when using these lateral cues. Each of the reaction time × location curves had a fairly abrupt decrease lateral to the cued location, and a gradual taper in the direction of true midline. Note that the decrease in reaction times lateral to the cued location suggests a distance effect, and is not a byproduct of lower spatial acuity at lateral locations. Spatial acuity continues to decline lateral to ±18°, in which case slower reaction times would have been expected.

## Discussion

The main finding across experiments was that when acoustic numbers are presented near the midline participants mislocalized sound location on the basis of number magnitude. Mislocalization was systematic, as the smallest numbers biased spatial perception towards left sided judgments, while increases in number magnitude were accompanied by having an increasingly right sided bias. In the left/right task this meant that as more space was judged to be on the “left” for small numbers the PSE was biased towards the right, and vice versa for larger numbers. This pattern of left-right biases near the midline was seen for different sets of numbers (1, 3, 8, 10 in Experiment 1; 1–9 in Experiments 2–4), in two different languages (German, English), and with two different methods to assess spatial judgments (pointing, left/right judgment). The location of the previous number in the left/right task also biased spatial judgments of the next number, irrespective of magnitude and whether or not a cue was given between numbers. Lastly, reaction times also had a distance effect centered on the midline location, and when cued for ±18° the peak of the distance effect was shifted towards the true midline for both left and right side cues.

The effect sizes of the number biases on perceptual measures were large, but the absolute magnitude of systematic perceptual bias was small (~1-2°). A small absolute bias would make sense because large biases would lead to illusory shifts in the perceived sound location even if the source is stationary. The main value of our findings is in terms of theory, with the implication that long-term knowledge can influence perception at a very basic level. An open question is whether the present results are due to bias in attention or perception. Both concepts are useful, but are nonetheless strongly, and perhaps inseparably, linked. We report here initial observations to define the phenomena of number biases on spatial hearing. Thus additional work is needed to define the role of attention. The main reason that, as a starting point, we are interpreting the findings in terms of a perceptual bias rather than an attentional bias is that our experiments used methods that are widely used to study perception.

### Number-related spatial biases: stimulus and task factors

We first consider whether the physical features of the sounds could account for the findings. In terms of overall precision in sound localization, Experiments 2–4 presented stimuli near midline, which has the highest acuity, while the lateral locations in Experiment 1 would have lower acuity^43^. Speech sounds have a variety of features such as the high-frequency noise-like sibilant fricatives at the beginning of the English words “six” and “seven”, which are acoustically quite different from the stop consonant /t/ at the beginning of “two” or the long diphthong /aI/ at the beginning of the German word “eins”. These differences in frequency bands might differentially relate to spectral cues used to localize sounds in the horizontal plane. It has long been known^44^ that the perceived location of a fixed pure-tone or narrow-band sound source can change considerably when its frequency varies (for review, see ref.^43^). Usually, these effects result in underestimation or overestimation of sound eccentricity, even though slight systematic errors to the left or right can also occur in the midline range (see Fig. 2.3 in ref.^43^). It is not clear how different sound features of numerals would lead to left/right biases in localization. Because of the well-known dominance of spatial cues at stimulus onset for sound localization^45^, the reverse control stimuli used in Experiment 1 only partially took into account this issue. However, there was no systematic relation between the spectral content of numeral words and number magnitudes, thus arguing against the possibility that the present findings can be explained by physical factors (see Supplemental Figs 1 and 2). A systematic bias in sound features with respect to number magnitude also goes against the conventional notion in linguistics that the sound of most words is arbitrarily mapped onto meaning^46^. Another consideration against a physical feature explanation is that similar results were seen using different languages (Experiments 1 vs. 2–4). Lastly, there were different patterns of results among the midline and lateral cueing experiments that used the same speech stimuli. Taken together, it is rather unlikely that the findings can be explained by physical features of the sounds that happen to covary with number magnitude.

### Long-term memory influences on attention and perception

We have emphasized the distinction between situations where information in long-term memory affects performance and instances where long-term memory has an influence on the subject’s perceptual experience. Since the advent of cognitive psychology there have been many observations of long-term memory’s influence on attention and performance, such as hearing one’s name in an unattended channel during dichotic listening^47^, seeing a previously reinforced stimulus^48^, and learning the significance of spatial locations and the broader context^45,46^. Studies have tested whether number magnitude implicitly shifts spatial attention to the left (smaller numbers) or right (larger numbers), but the results have been mixed^51,52^. There is also a large literature on spatial aspects of language, such as using space as a metaphor when expressing time in terms of distance, but this literature also refers to processes beyond basic perception^53^. As discussed in the introduction, the SNARC effect revealed that number magnitude can influence reaction time, which largely reflects response selection processes^30^. For completeness, we note that there are other influences on the SNARC effect, such as subject strategies^54^.

In contrast, only a few studies have examined long-term memory influences on the perceptual experience. For judgments of color it was found that typical colors of objects can bias color judgments^10,11^, and memories of musical key can influence pitch judgments^13^. The current results provide further support for the idea that long-term memory can directly bias perception, and expands this work into the realm of spatial hearing. In addition, the current study differed from prior efforts by observing a spatial bias even when the meaning of the words (numerosity) did not have an obvious relation to the nature of the perceptual bias (spatial). This makes it less likely that subjects were subtly influenced by task demands, as a left to right number line is commonly used in Western schools to help children learn number magnitudes.

The motivation for hypothesizing that numbers would impart a spatial perceptual bias was supported by multiple lines of work on the relations between numbers and space^22^. Studies of the time course of lexical access, which maps perceptual features onto meaning, show that neural responses based on meaning occur within ~200–400 ms when using intracranial recordings^55,56^ or scalp-recorded EEG^57,58^. Within this same time period recent studies have shown that auditory spatial coding develops based on sound location^59^ and sound location relative to an attended location^60,61^. Thus the timing of early lexical access and auditory spatial representations were congruent, and occurred well before subjects made their response in our experiments. Convergent evidence is provided by a crossmodal task where subjects viewed numbers and rated the loudness of simultaneous sounds^33^. This report not only found an influence of seeing numbers on loudness perception, but also observed that the bias was only evident when visual and auditory stimuli were presented at the same time.

From a broader perspective, the current results may connect to theories of semantic memory that stipulate that concepts include representations of lower-level sensory features, which is one variant of embodied cognition^62^. Spatial coding of numbers usually does not literally relate to differences in stimulus features, but instead may be associated with number ordering experiences within cultures having a convention for spatial number order such as a number line^16^. Alternatively, the connection between numbers and space that seems critical to the spatial biases we observed may reflect a system for representing analog magnitudes, which include space, numbers, and time^63^. Future work would be needed to explore these possibilities.

The role of short-term memory is implicit in this study, as well as others, because a certain range of numbers is given during the experiment, and number magnitudes are defined relative to this range. Similarly, in a reaction time task instructions to participants on how to visualize numbers (either as a ruler or analog clock face) led to opposite relations to space because small magnitudes are on the left of a ruler but on the right side of the clock^54^. The specific role and mechanisms of short-term memory in numeric studies is controversial. However, a more recent proposal that numeric bias reflects a combination of ordinal information in long-term memory and that short-term memory imparts the spatial correlate has been gaining support^64–66^. The present study was not designed to distinguish among the possibilities, but future work along these lines may help to better define the role(s) of memory codes in numeric bias. Instead, the main focus here was on the fact that long-term memory concerning number magnitudes and/or their ordering would be required for the acoustic stimuli to impart any perceptual bias on the basis of numerosity.

### Eye position and sound localization

Eye position can influence sound localization. Most reports showed localization shifts opposite to the direction of gaze^67–70^, but some found localization shifts that developed over time in the same direction of gaze^71,72^. Any eye position effects in the current study would only be a source of additional error if they were unrelated to number magnitude. However, a recent study showed a tendency for eye movements to the left after subjects heard small numbers, and movements to the right for larger numbers (experiment 2 in^73^). There were clear effects at a latency of ~1,600 s, and small, brief, effects at ~800 ms. Importantly, the subjects in Experiments 2–4 of the current study, on average, responded much earlier than the substantial effects from eye position reported in^73^ (Experiment 2: 545 ± 28 ms; Experiment 3: 639 ± 28 ms; Experiment 4: 762 ± 24 ms). In addition, work using a psychophysical method similar to Experiment 2 in this study found no consistent eye-position bias in a left/right judgment task with reference to the subjects’ subjective straight ahead (Experiment 1B in Lewald, 1997^74^). In a pointing task, localization biases were in the opposite direction of eye position^68,69^, which would result in an underestimation of the effect of number magnitude bias on sound localization when pointing. Taken together, eye position was unlikely to have affected the main results and conclusions of this study, but examining relations between eye position and number cognition in visual and auditory modalities is an interesting avenue for future research.

### Differences when stimuli are near the midline vs. lateral locations

When a cue was used to define a midpoint for left/right judgments, as in Experiments 3 and 4, the number bias was greatest when cueing on the left, moderate but statistically comparable to the no-cue condition at midline, and was not evident when the cue was on the right side. For the left cue the number magnitude bias was compressed to include the numbers “one” up to approximately “two” or “three”, after which there was a general bias toward the midline for the remaining numbers (Figs 3A and 4A). In conjunction with the irregular PSE pattern in the right cue condition, these findings suggest that prominent “number line-like” effects seen here are not due to the subject demand, such as consciously trying to make their spatial judgments about sound location match the order of a number line. The number magnitude effects were very large in the left cueing condition, and we speculate that they may relate to an interaction between spatial cueing and a potential logarithmic representation/compression of number magnitude suggested by previous work^75^.

### Reference frames for spatial biases and the two channel model of sound localization

The present study found two instances of spatial biases that were evident in the central-eccentric direction, indicating a spatial reference frame in the direction towards or away from the midline axis. First, the curves of reaction time vs. location were symmetrical about midline in Experiments 2 and 3, but when either left or right lateral cues were used in Experiment 4 the portion of the curve with the longest reaction times was skewed towards midline. This distortion in the distance effect was likely due to both the true midline and the cued location having an influence on reaction time. The second instance was in Experiment 1, where localization judgments of sounds perceived as speech had an eccentric bias relative to when the same sound was played in reverse. A similar eccentric bias was seen for perception of white noise after presentation of an acoustic number at the same location, particularly for locations of at least ±18° from midline^32^.

We speculate that the spatial biases near midline, which tend to be in the left-right direction, and more lateral locations, which tend to have central-eccentric biases, may reflect the dynamics of the two-channel model for sound localization. In brief, the two-channel model proposes that sound is represented in the auditory system by two channels, with each tuned to one side of the midline plane^76,77^. Sound location would be represented by an analog value of the difference between the two channels. The two channels are maximally activated for lateral sounds, and have decreasing activation that slopes downward and crosses at the midline. Recently, there is growing experimental evidence from neuroscience studies supporting the “opponent-channel model”, which may be related to this view (for review, see ref.^78^). The channels overlap at midline, and each is somewhat responsive to a small part of the opposite hemispace. Behavioral studies have long shown that locations near midline have the best spatial resolution^79^. This precision would be supported by the difference between the two channels where the slope is greatest, also near midline^77^. We speculate that this region of overlap between the channels flanking midline is conducive to coding number magnitude in the left-right direction. Coding of more lateral locations, beyond ~±18–30° would largely be represented by one of the channels, which progressively increases in output from the central to eccentric direction in each hemispace.

Another intriguing possibility is that there is a third channel centered at midline, and it is activity in this channel that is related to number magnitude. Although the majority of auditory cortical neurons have broad responsiveness to sounds in contralateral space, there are a minority of neurons that are tuned to midline locations^80–83^. Recent behavioral^84–86^ and electrophysiological^87^ studies in humans have also supported the idea of a midline channel. The different patterns of results for stimulus locations near midline vs. more eccentric locations in this study as well as a previous report^32^ are consistent with a form of number bias imposed by a midline channel that differs from that of lateral channels. However, this is speculative because the possibility of a third channel in humans is not well-established. To our knowledge this report and our previous publication^32^ are the first to show number biases on spatial hearing. Further investigation into the reasons and mechanisms for these memory biases on spatial hearing may provide constraints to more general theories of spatial hearing.

### Sequence effects

When subjects made left/right forced-choice judgments on stimuli that were located in the median sagittal plane (0°) there was a strong bias away from the side of the previous number. Although percentage of “right” judgments was measured rather than PSE, this may nonetheless reflect a shift in PSE towards the prior number’s location. A shift in PSE to the prior number’s location would explain why left/right judgments at 0° are opposite to the side of the prior number, as a PSE shift to the left means that the right side of the PSE would rotate over to the true 0° location. Note that this bias was present even when a white noise cue was presented in between the two numbers. Subjects did not make left/right judgments of the cues, so it is unknown whether the previous number location biases judgments of both the next cue and number. The sequence effect may be related to the auditory Roelofs effect (for the visual Roelofs effect see ref.^88^), in which an auditory target stimulus is perceived as shifted away from a simultaneously presented background sound^89^. A similar effect has been demonstrated for successive presentation of the target and the background, i.e., when the target stimulus was presented 1 s after the offset of the background sound^90^.

The sequence effects here differ from previous studies of sequential localization biases that used longer periods of stimulus adaptation (10 s of seconds, e.g. ref.^91,92^), simultaneous targets and adapters^93^, or very short intervals between adjacent stimuli^94^. Sequence effects on perceptual judgment at comparable inter-stimulus intervals have been observed when making loudness judgments^95^, and may relate to shorter-lived attentional repulsion effects observed in the visual modality^96,97^. Lastly, we note that the observed sequence effects are really a family of functions. Sequence effects can be measured at any of the locations used to define the psychometric function. Only midline judgments were presented here because the ambiguity of 0° was a useful way to detect left/right biases, and the main goal of the study concerned number magnitudes rather than sequence effects.

### Conclusion

The present four experiments demonstrated a clear bias of number magnitude on sound localization. Especially when the acoustic numbers were presented near the midline, participants perceived the sound location as being shifted towards the left for small numbers and towards the right for large numbers. The results suggest an analog relation between representation of number magnitude and spatial coding in the auditory modality, and indicate that semantic information in long-term memory can influence a basic aspect of auditory perception. These findings complement studies in the visual modality that did not directly assess perception; studies that also show that in Western number systems smaller numbers are associated with the left side of space and larger numbers to the right^14,15,22^.

## Electronic supplementary material

Supplementary information
