# Supplementary material for: Numerical value biases sound localization
Source: Sci Rep. 2017 Dec 8;7:17252. doi: 10.1038/s41598-017-17429-4 (PMC5722947; doi:10.1038/s41598-017-17429-4)
Supplement: Supplementary file 1 — Supplementary information [file 41598_2017_17429_MOESM1_ESM.pdf]

Numerical value biases sound localization

Edward J. Golob[1,2,5], Jörg Lewald[3,4], Stephan Getzmann[3,4,]

Jeffrey R. Mock[1,5]

[1] Department of Psychology, Tulane University, New Orleans, LA, USA

[2]Program in Neuroscience, Tulane University, New Orleans, LA, USA

[3] Faculty of Psychology, Ruhr University Bochum, D-44780 Bochum, Germany

[4]Leibniz Research Centre for Working Environment and Human Factors, Ardeystrasse 67, D-44139 Dortmund, Germany

[5]Present address: Department of Psychology, University of Texas, San Antonio

Correspondence:
Edward J. Golob, PhD
Department of Psychology
1 UTSA Circle
University of Texas, San Antonio,
San Antonio, TX 78249
email: edward.golob@utsa.edu

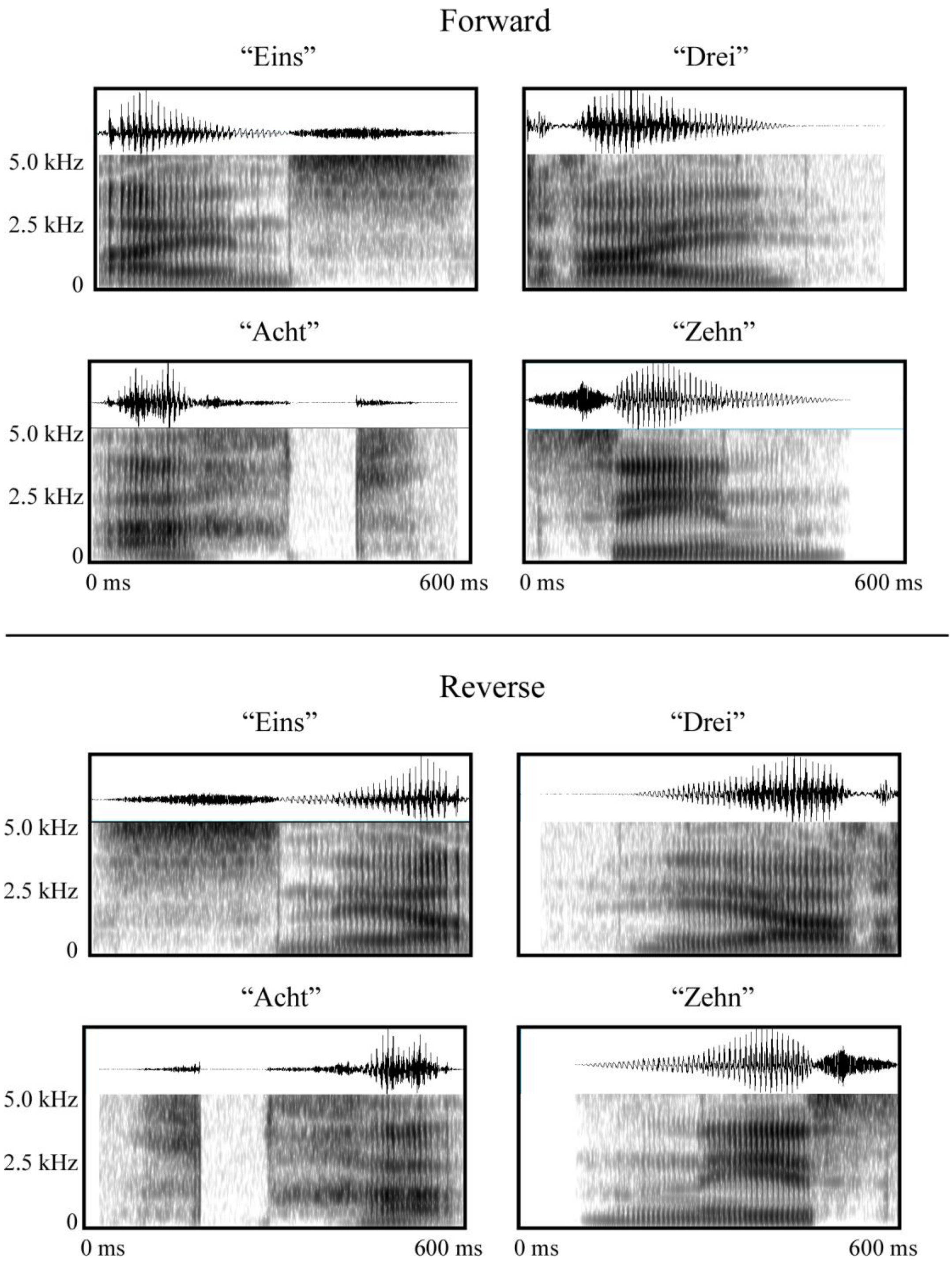

Supplemental Figure 1. Plots of speech waveforms and spectrograms for number stimuli used in Experiment 1. Forward presentation is in the top panel, and reverse presentation is shown in the lower panel.

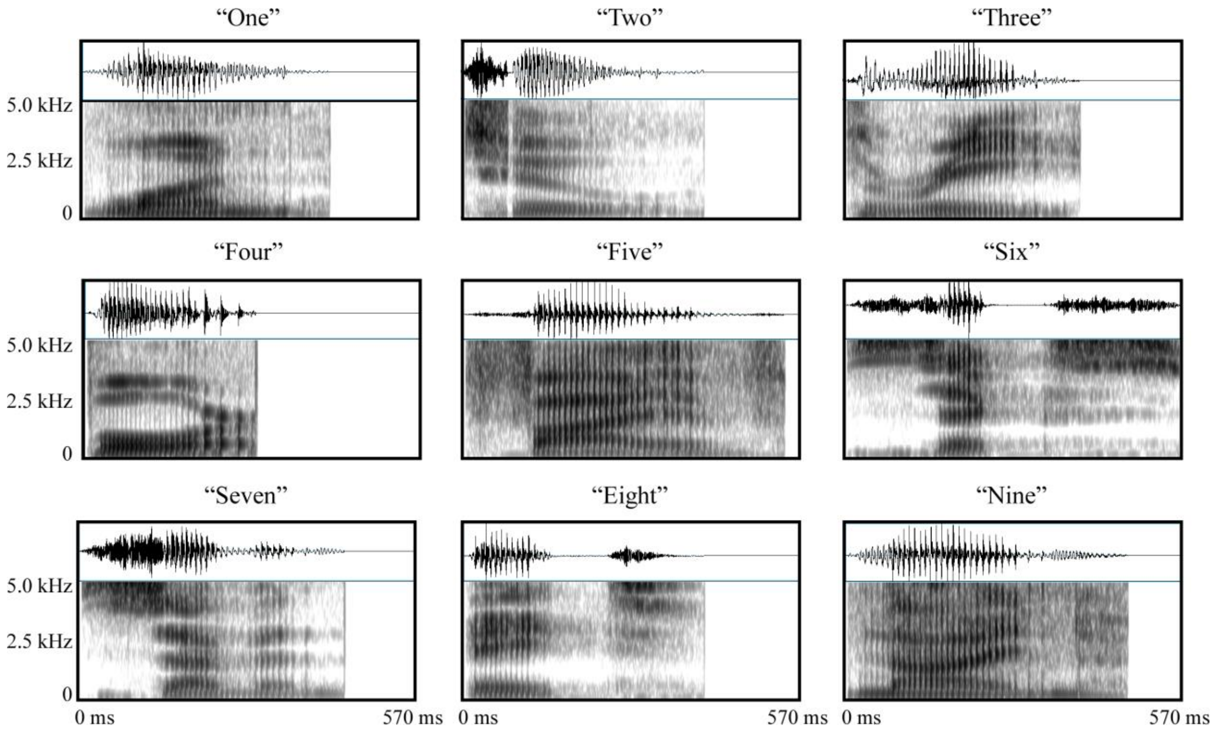

Supplemental Figure 2. Plots of speech waveforms and spectrograms for number stimuli used in Experiments 2, 3, and 4. Stimuli were from the 0° (midline) virtual location.
